# The interdependence of cigarette, alcohol, and marijuana use in the context of school-based social networks

**DOI:** 10.1371/journal.pone.0200904

**Published:** 2018-07-20

**Authors:** Cheng Wang, John R. Hipp, Carter T. Butts, Cynthia M. Lakon

**Affiliations:** 1 Department of Sociology, University of Notre Dame, Notre Dame, IN, United States of America; 2 Department of Criminology, Law and Society, University of California, Irvine, Irvine, CA, United States of America; 3 Department of Sociology, University of California, Irvine, Irvine, CA, United States of America; 4 Department of Statistics, University of California, Irvine, Irvine, CA, United States of America; 5 Program in Public Health, University of California, Irvine, Irvine, CA, United States of America; Harvard Medical School, UNITED STATES

## Abstract

The concurrent or sequential usage of multiple substances during adolescence is a serious public health problem. Given the importance of understanding interdependence in substance use during adolescence, the purpose of this study is to examine the co-evolution of cigarette smoking, alcohol, and marijuana use within the ever-changing landscape of adolescent friendship networks, which are a primary socialization context for adolescent substance use. Utilizing Stochastic Actor-Based models, we examine how multiple simultaneous social processes co-evolve with adolescent smoking, drinking, and marijuana use within adolescent friendship networks using two school samples from early waves of the National Longitudinal Study of Adolescent to Adult Health (Add Health). We also estimate two separate models examining the effects from using one substance to the initiation and cessation of other substances for each sample. Based on the initial model results, we simulate the model forward in time by turning off one key effect in the estimated model at a time, and observe how the distribution of use of each substance changes. We find evidence of a unilateral causal relationship from marijuana use to subsequent smoking and drinking behaviors, resulting in the initiation of drinking behavior. Marijuana use is also associated with smoking initiation in a school with a low substance use level, and smoking cessation in a school with a high substance use level. In addition, in a simulation model excluding the effect from marijuana use to smoking and drinking behavior, the number of smokers and drinkers decreases precipitously. Overall, our findings indicate some evidence of sequential drug use, as marijuana use increased subsequent smoking and drinking behavior and indicate that an adolescent's level of marijuana use affects the initiation and continuation of smoking and drinking.

## Introduction

The concurrent or sequential usage of multiple drugs during adolescence is a critical public health problem, spawning a large literature focusing on whether usage of one substance leads to usage of others [1–3]. The study of interdependence in adolescent substance use yields insight into potential patterns regarding which drugs are used sequentially or concurrently. As these risk behaviors co-occur and accumulate over time for certain individuals and social groups, there is potential to concentrate risk and negative sequelae among these concurrent users making concurrent users a high risk population that may be in need of prioritized and targeted intervention. In addition, to the extent that use of one substance affects the usage of another among adolescents, accounting for this interdependence in substance use is important as it can minimize the possibility of obtaining spurious relationships and possibly biased model estimates.

While some studies indicate that cigarette smoking is a strong predictor of the concurrent or subsequent usage of alcohol and marijuana [4–7], other studies find that alcohol use increases the likelihood of cigarette smoking [8]. In other research, a mutually reinforcing relationship was detected between adolescent alcohol use and smoking [9], and between cigarette smoking and marijuana use during adolescence and young adulthood [10–13]. In contrast, other research found that previous use of alcohol did not predict the initiation of marijuana use [14–15].

Existing studies also indicate that adolescent users of marijuana frequently smoke cigarettes, either as a substitute when marijuana is scarce, or as a means of counteracting the sedating effects of marijuana [11, 16]. The complementary usage of tobacco and marijuana in adolescence may contribute to the eventual dependence on nicotine [13, 17–18]. The complementary usage of these two substances might be the greatest public health consequence from marijuana use in adolescence [18].

There may also be complementarity in the usage of marijuana and alcohol. The observed high correlation of alcohol and marijuana use may be due to a shared genetic risk for drug use [19]. On the other hand, this observed correlation may be due to substituting one substance for another as a means of minimizing marijuana withdrawal symptoms [20]. In one study, daily marijuana users who underwent a period of abstinence drank more often if they had a previous diagnosis of alcohol abuse or dependence [20].

The importance of peers in the transmission of substance use behavior within adolescent friendship networks has given rise to a body of literature which focuses on how social networks can spread substance use behavior. These studies focus on how the co-evolution of adolescent friendship networks and substance use gives rise to peer influence (e.g., the process in which an adolescent aligns his or her behavior with that of peers) and selection (e.g., the social process whereby an adolescent chooses to associate with others who display a specific behavior or trait) effects within adolescent networks regarding a specific substance use behavior (e.g., smoking, drinking, or marijuana use). Peer influence is a type of social influence, and the latter has been theorized from numerous points of view including the *Dynamic Social Impact Theory*, which states that individuals will become more like those who are socially proximal and as a result their attributes will be correlated [21].

Given the importance of concurrent or sequential usage of cigarettes, alcohol, and marijuana, the purpose of this study is to examine the co-evolution of use of these substances within the dynamic landscape of adolescent friendship networks, which are a primary socialization context for adolescent substance use. A key methodological and theoretical challenge herein is that the context of peer networks must be taken in consideration when studying adolescents' interdependent substance use, because interpersonal association via peer influence or friendship selection likely shapes the concurrent or sequential use of substances. Not taking into account such peer network effects can result in biased estimates of the interdependence of substance use behaviors, or likewise, the effects of network influence.

**Fig 1:**
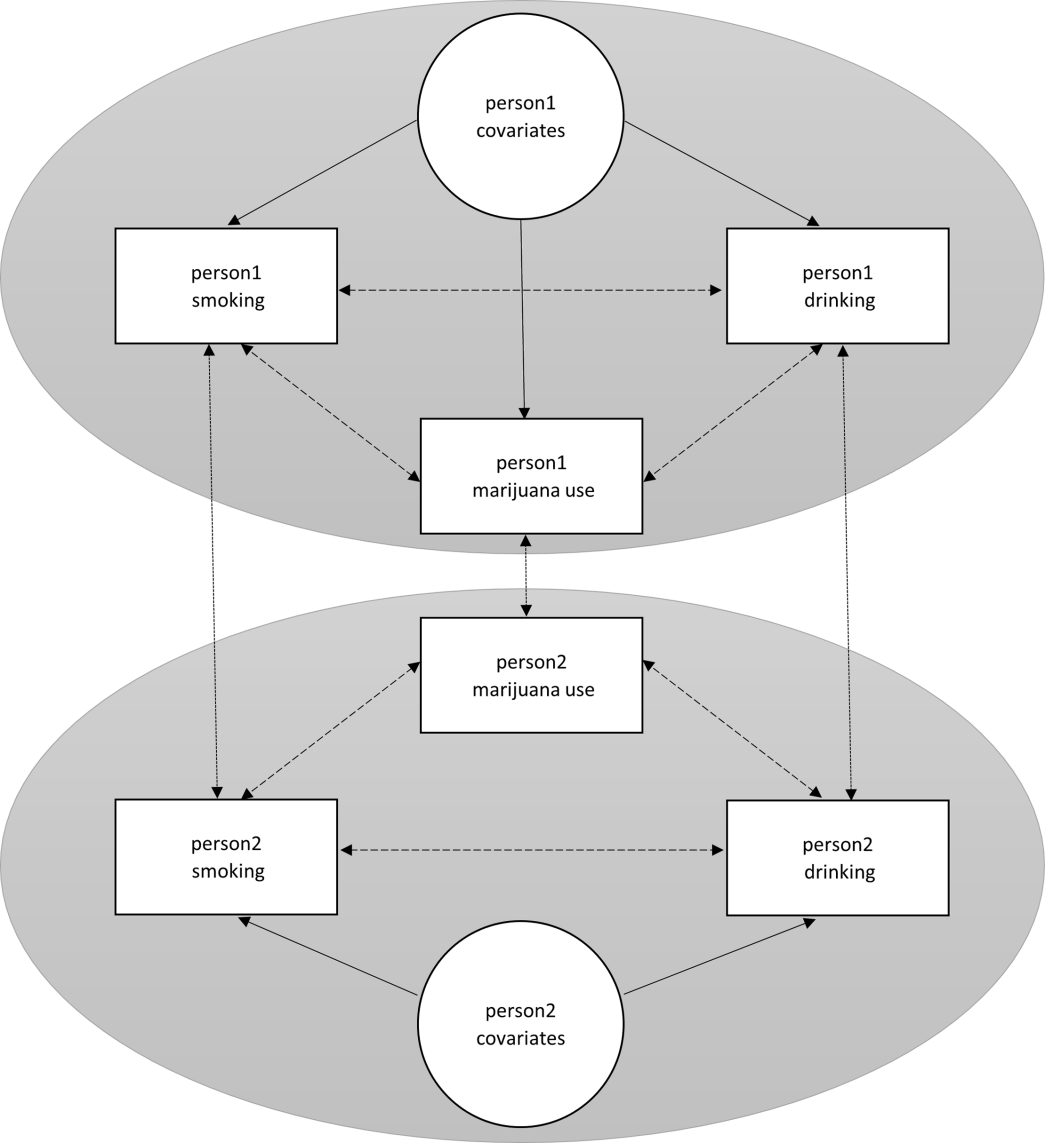
Multiple relationships in a hypothetical simple 2-person world of three substance use behaviors.

Fig 1 displays a hypothetical simple 2-person world in which the "true" model are the solid lines and the dashed lines show possibly spurious intra-personal effects of one substance use on another. This figure is informed by the *Dynamic Social Impact Theory* [21], as we posit that person 1 will become more like his or her peer, person 2, because of peer influence via modeling, shared opportunities, social proximity and the consolidation of attitudes and behaviors that may take place in adolescent friendship networks. This social process is captured in pathways from person 1 smoking to person 2 smoking, and in addition from person 1 drinking to person 2 drinking. Note that certain person-specific covariates affect an adolescent's usage of each substance: this will therefore lead to a correlation in usage across substances for the person. Not accounting for the across-person effects as shown in dotted lines–for example, how the smoking behavior of person 1 affects the smoking behavior of person 2 through a social influence or a selection effect–will result in these correlations being inappropriately captured by the dashed paths. Such correlations would be spurious, thus highlighting why it is critical to account for these network effects when studying concurrent substance use behavior. Although we do not show them here (for clarity), this figure could also represent pathways linking person 1 smoking and person 2 marijuana use, and analogously, person 1 drinking and person 2 marijuana use. These pathways may be a result of normative processes. The *subjective norms* construct from the *Theory of Planned Behavior* [22], which is the composite of the belief about whether most people approve or disapprove of a behavior and their corresponding motivation to comply with those important referents in their social environment, informs these normative pathways in our model. Adolescents who are smoking cigarettes, may through such normative beliefs, reinforce the use of other substances among their friends as they display pro-substance use norms and thus their friends may be motivated to comply with their attitudes and behavior, which would indirectly increase friends' acceptability of using marijuana or alcohol.

We are aware of just two existing studies that have simultaneously studied adolescent social networks and the use of more than two substances [23–24]. One longitudinal study focused upon smoking, drinking, and marijuana use in a sample of 129 Scottish youth (aged 13 to 15 years old) finding that while there were statistically significant peer influence effects on alcohol and marijuana use but not on smoking behavior, marijuana users smoked cigarettes more over time [23]. However, the other study, detected neither interdependent association effects nor peer influence effects on cigarette, alcohol, and marijuana use behaviors in a longitudinal study of a sample of US school students [24]. Note that the first study [23] utilized a relatively small sample and the second study [24] is limited to two waves of data, thus diminishing the statistical power of each study.

Finally, social influence may not necessarily have symmetric effects on initiation and cessation of substance use. For example, Haas and Schaefer [25] explored this idea in the context of smoking, and found some evidence that influence effects may have a stronger effect on starting smoking behavior, but weaker effects on stopping it. Although we do not have specific hypotheses regarding how such influence effects might operate for other substances such as alcohol or marijuana use, we nonetheless test this asymmetry possibility here in our analyses.

Building on past studies focusing on concurrent or sequential substance use in adolescence, we examine the co-evolution of adolescent friendship network ties and whether there was interdependence in usage of cigarettes, alcohol, and marijuana among 3,128 adolescents in two large schools. We utilize three waves of social network data from the National Longitudinal Study of Adolescent to Adult Health (Add Health) [26]. Ecological models of human development [27] informed the conceptualization of this study by situating adolescents in key social contexts exerting primary socialization forces including peer selection, peer influence, and parental influences. This study is also informed by the *Dynamic Social Impact Theory* [21], which forms the basis for why youths' behaviors will be correlated. Lastly, normative constructs from the *Theory of Planned Behavior* [22] guide our examination of the normative model pathways under study.

## Materials and methods

### Data

The data utilized in this study come from early waves of the Add Health study. The respondent record/information was anonymized and de-identified prior to analysis. This study was reviewed and granted approval under exempt review by the Institutional Review Board at the University of California, Irvine. This study does not employ human subjects directly, as our analyses utilize secondary data, which are de-identified. Add Health participants provided written informed consent for participation in all aspects of Add Health in accordance with the University of North Carolina School of Public Health Institutional Review Board guidelines that are based on the Code of Federal Regulations on the Protection of Human Subjects 45CFR46: http://www.hhs.gov/ohrp/humansubjects/guidance/45cfr46.html. Written informed consent was given by participants (or next of kin/caregiver) for their answers to be used in this study.

We construct separate samples for the two large saturation sample schools, one suburban Northeast public high school (*n* = 2,104) referred to as "Sunshine High" [28], and one rural Midwest public high school (*n* = 1,024) referred to as "Jefferson High" [29]. Our data come from the Add Health In-School Survey (conducted in October 1994 at Sunshine High and November 1994 at Jefferson High), the wave 1 In-Home Survey (conducted between May and November of 1995 in our two schools), and the wave 2 In-Home Survey (conducted between April and August of 1996 in our two schools) [26]. Therefore, the average time spans between wave 1 and wave 2 are 8.8 and 7.7 months for students in Sunshine High and Jefferson High, respectively. The average time spans between wave 2 and wave 3 are 10.9 and 11.1 months for students in Sunshine High and Jefferson High, respectively.

### Methods

We utilize the R-based Simulation Investigation of Empirical Network Analysis (RSiena) software package [30] to estimate Stochastic Actor-Based (SAB) models [31, 32]. We specify each model with three behavior equations in which we focus on how usage of one substance is influenced by the usage of the other two substances, along with one network equation in which we model the network evolution in tie formation and dissolution among adolescents in the school. We estimated the model separately on each school. Besides the key mechanisms illustrated in Fig 1, we adopt a forward selection approach for each parameter via score-type test [30]. In the behavior equations, the linear and quadratic effects capture the time trend of each substance use behavior; peer influence effects are measured as the sum of negative absolute difference between ego's and alters' behavior averaged by ego's out-degree. Additional covariates such as in-degree, parental support, parental monitoring, race (Sunshine High only), and depressive symptoms are added given that they have been shown to be important covariates in the existing literature, and given that the results from score-type tests reject the null hypothesis that their parameters are 0s. In-degree is important to test, given the debate in the existing literature about the importance of network centrality, or popularity, for explaining substance use [33]. We also control for the effects of how ego's use of one substance was influenced by alters' use of two other substances. In the network equation, we include endogenous network effects (e.g., reciprocity, triad closure, degree assortativity) and homophily selection effects for each substance use behavior as well as additional covariates such as race (i.e., Sunshine High only), gender, grade, and parental education as the results from score-type tests suggest to do so. 501 students in Sunshine High and 166 students in Jefferson High were 12th-graders at *t*_*1*_ and *t*_*2*_ (i.e., In-School Survey and wave 1 In-Home Survey) and graduated at *t*_*3*_ (i.e., wave 2 In-Home Survey). These 667 students were constructed as structural zeroes in the networks during the last wave. Due to a survey implementation error in Add Health, some adolescents could only nominate *one* female and *one* male friend at *t*_*2*_ (i.e., wave 1 In-Home Survey) and *t*_*3*_ (i.e., wave 2 In-Home Survey). We account for this with a limited nomination variable (measured as: -1 = changed from full to limited nominations, 0 = no change, and +1 = changed from limited to full nominations) in the network equation.

A Method of Moments (MoM) [30] estimation is used to estimate the behavior and network parameters in each model so that the target statistics in behaviors and networks can be most accurately calculated. We assess satisfactory model convergence with criteria of *t* statistics for deviations from targets (i.e., ideally less than 0.10 for each parameter) and the overall maximum convergence ratio (i.e., ideally less than 0.25). The results of a post hoc time heterogeneity test for the models found no evidence that the co-evolution of substance use behaviors and friendship networks was significantly different across the two time periods, providing no indication of estimation or specification problems. We also perform goodness-of-fit testing for key network statistics in both schools, and display the results in the S1 File.

Besides the main SAB model for each school sample, we estimate ancillary models that test whether the interdependent effects are symmetric in increasing and decreasing substance use. This is accomplished by differentiating the "creation" function and the "endowment" function in RSiena [31, 34]. This technique has been applied to explore the asymmetric peer influence effect on adolescent smoking initiation and cessation [25].

### Dependent network and behavior variables

The dependent network variable, friendship tie choice, is based on the question asking adolescents to nominate up to five female and up to five male best friends.

The substance use dependent behavior variables are constructed from adolescents' self-reports of their smoking, drinking, and marijuana use levels. We define the smoking, drinking, and marijuana use response category levels based on four considerations: (1) the statistical distribution of each substance use behavior among US adolescent in the mid-1990s, (2) how each substance use behavior question has been asked in the Add Health survey, (3) assuring that there are not too few observations for statistical reasons at a particular value, and (4) the approach utilized in previous studies, including Pearson et al. [23] and Mathys et al. [24] The original questions with regard to substance use behaviors were "During the past twelve months, how often did you smoke cigarettes / drink beer, wine, or liquor?" at *t*_*1*_ (i.e., In-School Survey) and "During the past 30 days, on how many days did you smoke cigarettes / drink alcohol / use marijuana?" at *t*_*2*_ and *t*_*3*_ (i.e., wave 1 and wave 2 In-Home Survey). We categorize smoking behavior into four levels based on activity over the previous 30 days with 0 = "never", 1 = "1–3 days", 2 = "4–21 days", and 3 = "22 or more days", drinking behavior into 5 levels based on activity over the previous 12 months with 0 = "never", 1 = "1–2 days", 2 = "once a month or less (3–12 times in the past 12 months)", 3 = "2 or 3 days a month", and 4 = "more than 1 or 2 days a week", and marijuana use behavior into 3 levels based on activity over the previous 30 days with 0 = "never", 1 = "1–10 times", 2 = "more than 10 times".

**Fig 2:**
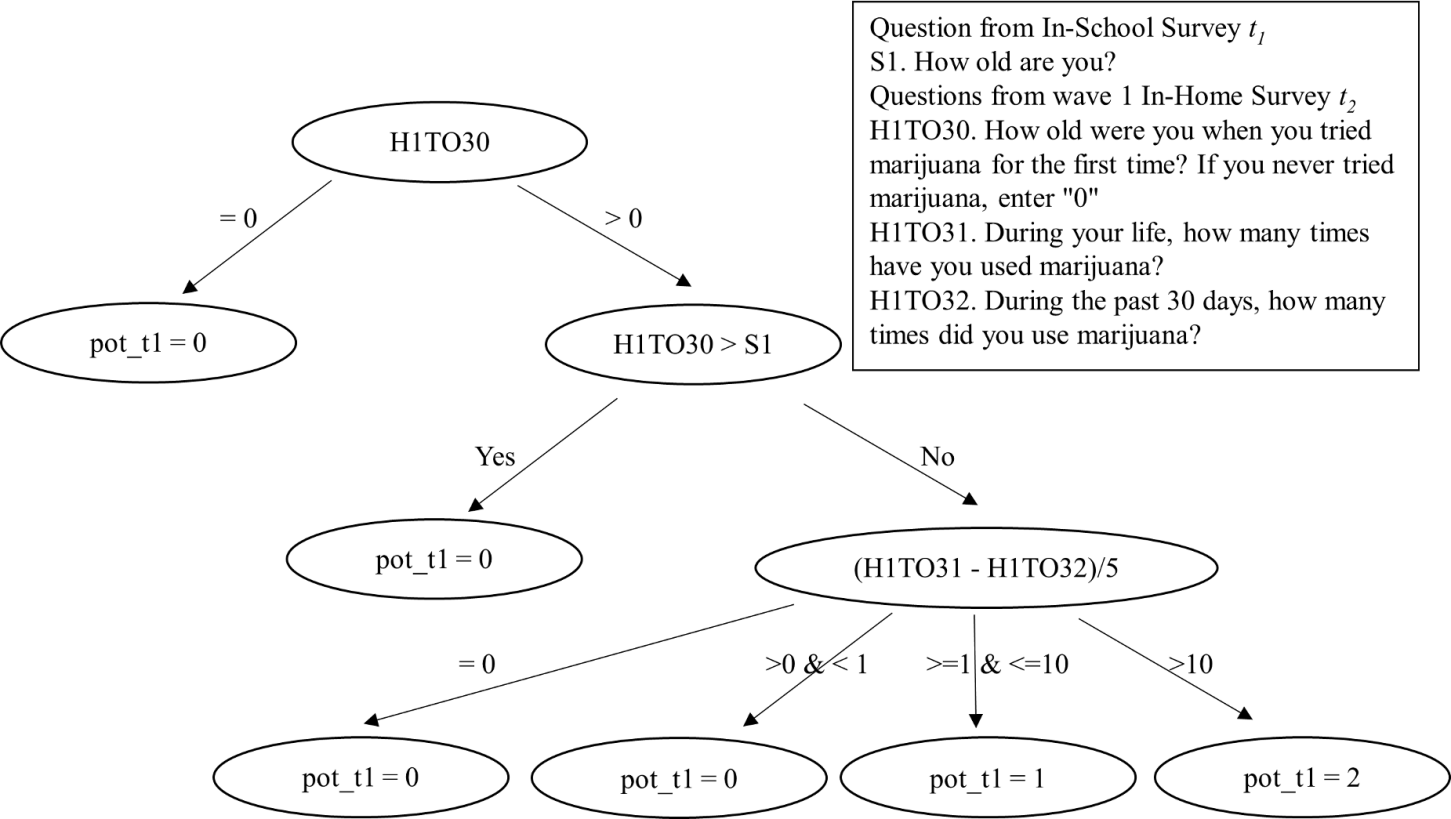
Reconstruction of marijuana use measure at *t*_*1*_ (In-School Survey).

A methodological challenge we face is that whereas the questions about smoking and drinking behavior were asked at all three waves, questions about marijuana use were only asked at *t*_*2*_ and *t*_*3*_ (i.e., wave 1 and wave 2 In-Home Survey). One approach would discard all the information at *t*_*1*_ (i.e., In-School Survey), but this strategy will reduce the efficiency of analysis, increase standard errors, and decrease statistical power. Instead, we reconstruct adolescent marijuana use at *t*_*1*_ based on four questions. Fig 2 provides a flow chart of the logic, and shows that we in fact have a considerable amount of information that can help us reconstruct probable values for the vast majority of the adolescents. First, if an adolescent has never tried marijuana at *t*_*2*_ (i.e., H1TO30 = 0 during wave 1 In-Home Survey), s/he would not have used it at *t*_*1*_, so we can safely code them as a zero at *t*_*1*_ (i.e., pot_t1 = 0; about 62% in Sunshine High and 55% in Jefferson High were in this scenario). Next, if an adolescent has tried marijuana at *t*_*2*_ but the age at which he or she tried was above his or her age at *t*_*1*_ (i.e., H1TO30 > S1), s/he would not have reported using it at *t*_*1*_, so we can safely code them as a zero at *t*_*1*_ (i.e., pot_t1 = 0; about 2% in Sunshine and Jefferson High were in this scenario). Finally, if an adolescent has tried marijuana at *t*_*2*_ and the age of usage was below his or her age at *t*_*1*_ (i.e., H1TO30 ≤ S1), we utilize information from two questions "During your life, how many times have you used marijuana?" (H1TO31) and "During the past 30 days, how many times did you use marijuana?" (H1TO32) at *t*_*2*_. In a few instances (about 1% of cases) the difference between these two variables is zero, which appears to be a reporting error as they reported all their usage in the last 30 days and yet that they started at a young age. We code them as a zero at *t*_*1*_ (i.e., pot_t1 = 0) under the presumption that this earlier usage was very limited, and perhaps experimental. However, if the difference is non-zero, since the In-School Survey was conducted at least six months before the wave-1 In-Home Survey, we divide this difference by 5 to average over five months [i.e., (H1TO31—H1TO32)/5]. Those with values less than 1 were categorized as non-users at *t*_*1*_ (about 15% in Sunshine High and 18% in Jefferson High were in this scenario), those with values between 1 and 10 were categorized as light users (pot_t1 = 1) and those with values above 10 were categorized as heavy users (pot_t1 = 2). Light users comprised about 16% of adolescents in Sunshine High and 17% of adolescents in Jefferson High. Likewise, heavy users comprised about 5% of the adolescents in Sunshine High and 8% of the adolescents in Jefferson High. Overall, this reconstruction strategy enabled us to estimate a three-wave SAB model for each of the two samples without discarding any data. The last step of the reconstruction procedure for the heavy marijuana users is not perfectly accurate and might mistakenly categorize a few light users as heavy users, since they could have used marijuana outside of the last five months. The proportion of cases that might have been misclassified is less than 10%. Furthermore, sensitivity tests in which the level of marijuana use for these uncertain cases was randomly assigned to "light" or "heavy" use exhibited similar results over a large number of samples (available upon request).

### Covariates

Our estimated SAB models include gender (0 = "male", 1 = "female"), grade (7th to 12th), race (only in Sunshine High), and parental education level (1 = "less than high school", 2 = "high school graduate", 3 = "some college or trade school", 4 = "graduate of college/university"). Depressive symptoms are included as a factor score based on 19 ordinal items modified from the Center for Epidemiologic Studies Depression Scale (CES-D; Cronbach's *α* = 0.87). Parental support and parental monitoring are constructed as standardized factor scores (means = 0, standard deviations = 1) through confirmatory factor analysis, with Root Mean Squared Error of Approximation (RMSEA) about .05 and Comparative Fit Index (CFI) greater than .95, which both suggest a good fit. Parental support is based on how adolescents rated their parents in 6 aspects: whether they communicated well, were "warm and loving", had a "good relationship" (1 = "strongly disagree" to 5 = "strongly agree" for the 3 items above), and whether the adolescents felt cared about, felt close (1 = "not at all" to 5 = "very much" for the 2 items above), and discussed personal problems with their parents (0 = "no", 1 = "yes"). Parental monitoring is based on 9 items: whether parents were home before school (0 = "never" to 4 = "always" with 5 = "took them to school"), after school (0 = "never" to 4 = "always" with 5 = "brought them home from school"), at bedtime (0 = "never" to 4 = "always"), present during dinnertime (0–7 days per week), and whether adolescents were allowed to decide their weeknight bedtime, weekend curfew, people they hung around with, and how much television and which television program they watched (0 = "yes" and 1 = "no" for 5 items above).

### Missing data

Regarding missing data, for students in Sunshine High the response rates were 76% (i.e., 1,600 out of 2,104) at *t*_*1*_ (i.e., In-School Survey), 82% (i.e., 1,721 out of 2,104) at *t*_*2*_ (i.e., wave 1 In-Home Survey), and 75% (i.e., 1,199 out of 1,603) at *t*_*3*_ (i.e., wave 2 In-Home Survey). In Jefferson High the response rates were 79% (i.e., 805 out of 1,024), 81% (i.e., 832 out of 1,024), and 74% (i.e., 635 out of 858) across the three waves. We imputed missing network data using the technique described in Wang et al. [35] given the evidence that failing to do so can result in in biased estimates [36]. Other actor attributes at *t*_*1*_ were imputed using the multiple imputation system of chained equations implemented in Stata. For the later waves, missing data is handled within the Stochastic Actor-Based models in RSiena software as suggested by Huisman and Steglich [37] and Ripley et al. [30]. The 501 and 166 students (for Sunshine and Jefferson High, respectively) who graduated at *t*_*3*_ and were no longer in the network are treated as structural zeros in the Stochastic Actor-Based models at the last wave.

## Results

### Descriptive statistics

Network statistics are measured at three waves. As shown in Table 1, in both school samples the number of out-going ties decreased over time due to limited nomination restrictions, graduation, moving, dropping out, and sample attrition/non-response/missing network data. The reciprocity index is the proportion of ties that were reciprocal. The proportion of reciprocal ties over all out-going ties was 4% to 10% higher in Jefferson High than in Sunshine High at each wave. The transitivity index is the proportion of 2-paths (ties existing between AB and BC) that were transitive (ties existing between AB, BC, and AC, which represent the dyadic relations among three students A, B, and C.), which is similar in the two schools. The Jaccard index measures the network stability between consecutive waves. There were substantial changes in friendship ties across waves, with the Jaccard index staying at .16 in Sunshine High and ranging from .21 to .22 in Jefferson High. Due to a survey implementation error in Add Health, some adolescents could only nominate *one* female and *one* male friend at *t*_*2*_ and *t*_*3*_ (i.e., wave 1 and wave 2 In-home Survey). Most limited nomination restrictions happened at wave 2, and involved less than 5% in the two schools.

**Table 1:** Descriptive statistics.

	Sunshine High (*n* = 2,104)			Jefferson High (*n* = 1,024)		
Network statistics	*t*_*1*_	*t*_*2*_	*t*_*3*_	*t*_*1*_	*t*_*2*_	*t*_*3*_
Out-going ties	5,685	4,201	2,296	6,063	3,713	2,484
Reciprocity index	0.30	0.28	0.25	0.34	0.35	0.35
Transitivity index	0.19	0.19	0.14	0.18	0.19	0.20
Jaccard index	0.16		0.16	0.22		0.21
Limited nominations (%)	0	3.49	1.52	0	4.82	0.41
**Smoking (past 30 days, %)**						
0 = never	68.60	78.28	71.76	42.01	53.17	45.39
1 = 1-3days	17.54	7.44	9.37	21.31	9.12	11.68
2 = 4–21 days	4.91	7.07	8.91	9.02	11.58	10.55
3 = 22 or more days	8.95	7.21	9.97	27.66	26.13	32.38
**Alcohol use (past 12 months, %)**						
0 = never	44.03	51.56	53.76	30.53	34.32	37.60
1 = 1 or 2 days	26.54	19.19	14.83	23.46	19.77	13.73
2 = once a month or less (3–12 times in the past 12 months)	8.59	12.35	11.11	12.70	18.55	15.98
3 = 2 or 3 days a month	6.66	6.06	6.80	13.63	14.34	14.04
4 = more than 1 or 2 days a week	14.19	10.84	13.50	19.67	13.01	18.65
**Marijuana use (past 30 days, %)**						
0 = never	83.05	73.00	62.63	77.15	69.77	61.07
1 = 1–10 times	7.76	14.78	20.71	10.14	18.03	20.49
2 = 11 or more times	9.18	12.21	16.67	12.70	12.19	18.44
**Response rate (%)**	76.05	81.80	74.80	78.61	81.25	74.01
**Female (%)**	47.52			48.46		
**Grade level (%)**						
7th grade	0.00			0.00		
8th grade	0.00			0.00		
9th grade	0.00			28.79		
10th grade	37.23			28.48		
11th grade	33.43			21.72		
12th grade	29.34			21.00		
**Parent education level (%)**						
Less than high school	21.67			5.23		
High school	30.62			38.32		
Some college or trade school	28.79			36.48		
Graduate of college/university	18.92			19.98		
**Depressive symptom, mean (SD)**	0.14(0.53)			0.00(0.53)		
**Parental support, mean (SD)**	-0.05(0.30)			-0.04(0.29)		
**Parental monitoring, mean (SD)**	-0.01(0.12)			-0.04(0.10)		

With respect to smoking behavior, there were between 69% and 78% non-smokers (with a value of 0) in Sunshine High over the three waves, and between 7% and 10% heavy-smokers (with a value of 3). In Jefferson High, there were between 42% and 53% non-smokers and between 26% and 32% heavy smokers. Sunshine High also had more non-drinkers than Jefferson High (about 50% vs. 34%), and more non-users of marijuana (about 73% vs. 69%).

The descriptive statistics of covariates are reported in the lower part of Table 1.

### The estimated SAB model

As shown in Table 2, our estimated SAB model includes a smoking behavior equation, a drinking behavior equation, a marijuana use equation, and a network equation.

**Table 2:** Stochastic actor-based models of substance use of cigarettes, alcohol, and marijuana and friendship networks with number of friends who smoked/drank/used marijuana effects.

Effect name	Sunshine High	Jefferson High
**Smoking behavior**	**beta** **(s.e.)**	**beta** **(s.e.)**
Rate smoking behavior (period 1)	13.18** (4.86)	9.14*** (1.57)
Rate smoking behavior (period 2)	21.31*** (3.28)	13.84*** (3.42)
Smoking behavior linear shape	-2.51*** (0.11)	-2.16*** (0.10)
Smoking behavior quadratic shape	0.73*** (0.04)	0.70*** (0.06)
In-degree	0.01 (0.01)	0.01 (0.01)
Smoking behavior peer influence	0.54*** (0.13)	0.77*** (0.15)
Parental support	-0.13 (0.19)	-0.02 (0.14)
Parental monitoring	-0.05 (0.26)	-0.05 (0.22)
Black	-0.33** (0.12)	-
Latino	-0.13* (0.05)	-
Depressive symptoms	0.07 (0.06)	0.12* (0.05)
Drinking behavior	0.05† (0.03)	0.02 (0.06)
Number of friends who drank	0.01 (0.02)	0.00 (0.01)
Marijuana use	0.22*** (0.06)	0.14* (0.06)
Number of friends who used marijuana	0.02 (0.05)	-0.02 (0.03)
**Drinking behavior**	**beta** **(s.e.)**	**beta** **(s.e.)**
Rate drinking behavior (period 1)	15.86*** (1.68)	9.94*** (0.94)
Rate drinking behavior (period 2)	16.83*** (2.24)	13.21* (5.68)
Drinking behavior linear shape	-1.38*** (0.07)	-1.01*** (0.11)
Drinking behavior quadratic shape	0.27*** (0.03)	0.20*** (0.02)
In-degree	0.01 (0.01)	0.01 (0.01)
Drinking behavior peer influence	0.28** (0.12)	0.38* (0.16)
Parental support	-0.06 (0.06)	-0.07 (0.06)
Parental monitoring	-0.19 (0.20)	-0.47** (0.17)
Black	-0.11* (0.04)	-
Latino	0.07† (0.04)	-
Depressive symptoms	0.06* (0.03)	0.02 (0.03)
Smoking behavior	0.03 (0.06)	0.01 (0.02)
Number of friends who smoked	0.01 (0.01)	0.01 (0.01)
Marijuana use	0.20*** (0.05)	0.15** (0.05)
Number of friends who used marijuana	0.01 (0.02)	-0.02 (0.01)
**Marijuana use**	**beta** **(s.e.)**	**beta** **(s.e.)**
Rate marijuana use (period 1)	2.68*** (0.50)	2.42*** (0.34)
Rate marijuana use (period 2)	4.97*** (0.57)	4.83*** (0.83)
Marijuana use linear shape	-2.48*** (0.29)	-2.14*** (0.32)
Marijuana use quadratic shape	1.13*** (0.13)	1.06*** (0.09)
In-degree	0.03 (0.05)	0.02 (0.02)
Marijuana use peer influence	1.43** (0.38)	1.32*** (0.49)
Parental support	-0.03 (0.23)	0.12 (0.19)
Parental monitoring	-0.34 (0.46)	0.00 (0.46)
Black	0.17 (0.11)	-
Latino	0.07 (0.09)	-
Depressive symptoms	-0.08 (0.08)	0.12 (0.12)
Smoking behavior	0.07 (0.11)	0.05 (0.06)
Number of friends who smoked	0.02 (0.06)	0.03 (0.05)
Drinking behavior	0.12 (0.08)	0.06 (0.08)
Number of friends who drank	-0.04 (0.04)	-0.04 (0.04)
**Friendship network dynamics**	**beta** **(s.e.)**	**beta** **(s.e.)**
Friendship rate (period 1)	16.04*** (0.81)	18.02*** (1.40)
Friendship rate (period 2)	7.75*** (0.78)	11.75*** (0.93)
Out-degree (density)	-4.15*** (0.18)	-2.14*** (0.40)
Reciprocity	3.11*** (0.16)	2.57*** (0.11)
Transitive triplets	0.71** (0.22)	0.62*** (0.05)
3-cycles	-0.29 (0.40)	-0.42*** (0.12)
Out-degree–popularity	-0.32*** (0.08)	-0.19*** (0.04)
In-degree–popularity	-0.05** (0.02)	-0.06** (0.02)
Out-out degree^(1/2) assortativity	-0.07* (0.03)	-0.12*** (0.02)
In-in degree^(1/2) assortativity	0.48*** (0.08)	0.37*** (0.07)
Race similarity	1.18*** (0.08)	-
Gender similarity	0.29*** (0.04)	0.23*** (0.06)
Grade similarity	0.62*** (0.05)	0.57*** (0.05)
Parental education similarity	0.09** (0.03)	0.05 (0.05)
Smoking similarity (peer selection)	0.01 (0.08)	0.24* (0.10)
Drinking similarity (peer selection)	0.12* (0.05)	0.13** (0.05)
Marijuana use similarity (peer selection)	0.27*** (0.07)	0.22* (0.09)
Limited nomination ego	-0.84*** (0.25)	-1.38*** (0.21)

Notes

† Two-sided p<0.1

* Two-sided p<0.05

** Two-sided p<0.01

*** Two-sided p<0.001

Based on the smoking behavior equation, those who were one point higher on the marijuana scale are 25% [exp(.22) = 1.25] and 15% [exp(.14) = 1.15] more likely to increase their own smoking behavior at the next time point in Sunshine High and Jefferson High, respectively. Those who drank alcohol did not smoke more over time. There is no evidence of cross-substance influence, as having more friends who drank or used marijuana did not impact a respondent's own smoking over time. In ancillary models, we measured *average* level of drinking or marijuana use for friends and these effects were also statistically insignificant. These results are shown in S1 Table. Regarding the other measures in the smoking behavior equation, we detect a negative smoking behavior linear shape parameter in both school samples along with a positive smoking behavior quadratic shape parameter. This suggests that adolescents were inclined to adopt lower levels of smoking behavior over time, but they also tended to stay as or become non-smokers or escalate to heavy-drinkers due to a pull towards extreme values of this scale. Turning to the peer influence effect, we find that adolescents' own smoking levels were affected by that of their best friends in both schools. There is no evidence that parental support or monitoring reduced levels of smoking over time in either sample. African Americans and Latinos smoked less than Whites in Sunshine High. Depressive symptoms were found to increase smoking behavior in Jefferson High.

In the drinking behavior equation, we find that an adolescent who was one point higher on the marijuana use measure was 22% and 16% more likely to increase their own alcohol use at the next time point in Sunshine High and Jefferson High, respectively. However, respondents' drinking was not related to their greater cigarette use. There is no evidence that friends' smoking behavior or marijuana use affected respondents' drinking behavior. This was the case whether measured as the number of friends who smoked or used marijuana, or as the average level of such behaviors. A negative linear shape effect and a positive quadratic shape effect are also confirmed regarding drinking behavior. An adolescents' drinking level was positively predicted by that of one's best friends. Whereas there is no evidence in these two networks that high levels of parental support impacted drinking levels of adolescents, we do see that higher levels of parental monitoring were associated with lower levels of drinking behavior over time in Jefferson High. In Sunshine High, African Americans were found to drink less than Whites, and depressive symptoms were found to increase drinking levels.

The marijuana use equation suggests no evidence that increasing usage of the other two substances leads to increasing marijuana use. We once again see no evidence of cross-substance influence, as the number of friends who smoked or drank or the average smoking or drinking level of friends is not related to ego's marijuana use levels over time. A negative linear shape effect and a positive quadratic shape effect are also detected on marijuana use behavior. Across both samples there is very strong evidence of a peer influence effect from an adolescent's best friends' marijuana use to an individual's own marijuana use. Higher levels of parental support or monitoring were not found to reduce levels of marijuana use over time. For all three substance use behaviors, there was no evidence that adolescents who are more "popular" (based on the number of network ties they have received) were any more likely to increase their substance use over time.

In the network equation the expected patterns are detected regarding the endogenous network structural effects across samples. At the dyadic level, adolescents did not randomly nominate peers as friends, since friendship ties inherently require the investment of time and energy, as indicated by the negative out-degree (density) parameters; instead, adolescents tended to nominate peers who had already nominated them as friends previously, as indicated by the positive reciprocity parameters. At the triadic level, adolescents tended to nominate a friend's friend as a friend but avoided ending in 3-person cyclic relationships. The negative out-degree/in-degree popularity parameters and the out-out degree (square root) assortativity parameters suggest that adolescents were less likely to befriend peers who have already made/received many friendship nominations or have similar out-degrees. Instead, they were more likely to befriend peers with similar in-degrees, as indicated by the positive in-in degree (square root) assortativity parameters. We also find that adolescents were more likely to nominate peers as friends if they were of the same gender, race (in Sunshine High), and grade. Grade is a particularly strong effect, as adolescents were 86% and 77% more likely to nominate a friend if they were in the same grade than if they were in a different grade in Sunshine High and Jefferson High, respectively. Lastly, the limited nomination parameter shows that for adolescents who encountered the administrative error of being limited to nominate only one male or one female friend, their odds of nominating friends is re-adjusted by the SAB models to be 132% larger in Sunshine High and 297% larger in Jefferson High than those with no such problem.

### Uptake and maintenance processes from marijuana use to smoking and drinking

Whereas our initial models tested the relationship between interdependent substance use behavior, they assumed that these effects are symmetric: that is, usage of one substance equally increases or decreases usage of another substance. In our next set of models, we relax this assumption and test whether usage of one substance increases behavior of another substance (termed "creation" in RSiena models) or decreases behavior (termed "endowment" in RSiena models), or both [30]. These models were estimated separately as the combined model exhibited extreme collinearity. As shown in Table 3, there is a significantly positive creation function from marijuana use to drinking in both samples, implying that respondents' marijuana use increased their odds of drinking initiation. Thus, one unit higher marijuana use made a non-drinker 62% and 60% more likely to start drinking rather than stay as a non-drinker at the next time point in Sunshine High and Jefferson High, respectively. On the other hand, the endowment function from marijuana use to drinking is not statistically significant at either school, implying that marijuana use does not affect the likelihood of stopping drinking behavior.

**Table 3:** Stochastic actor-based models decomposing interdependent substance use into increases (creation function) and decreases (endowment function).

Effect	Sunshine High	Jefferson High
**Smoking behavior**	**beta** **(s.e.)**	**beta** **(s.e.)**
Drinking behavior (creation function)	0.08 (0.13)	0.00 (0.07)
Marijuana use (creation function)	0.48*** (0.12)	0.24 (0.17)
**Drinking behavior**	**beta** **(s.e.)**	**beta** **(s.e.)**
Smoking behavior (creation function)	0.10 (0.20)	0.10 (0.07)
Marijuana use (creation function)	0.48* (0.23)	0.47*** (0.14)
**Marijuana use**	**beta** **(s.e.)**	**beta** **(s.e.)**
Smoking behavior (creation function)	0.10 (0.13)	0.08 (0.16)
Drinking behavior (creation function)	0.20 (0.15)	0.21 (0.17)
**Smoking behavior**	**beta** **(s.e.)**	**beta** **(s.e.)**
Drinking behavior (endowment function)	0.11 (0.08)	0.07 (0.08)
Marijuana use (endowment function)	0.20 (0.16)	0.24* (0.12)
**Drinking behavior**	**beta** **(s.e.)**	**beta** **(s.e.)**
Smoking behavior (endowment function)	0.02 (0.07)	-0.05 (0.06)
Marijuana use (endowment function)	0.22 (0.15)	0.13 (0.12)
**Marijuana use**	**beta** **(s.e.)**	**beta** **(s.e.)**
Smoking behavior endowment function)	0.09 (0.42)	-0.07 (0.19)
Drinking behavior (endowment function)	0.08 (0.35)	-0.17 (0.17)

Notes

1. Both models control for the same effects on substance use dynamics and network dynamics as included in the SAB models shown in Table 1. The parameter estimates and standard deviations are similar. The full results are available from the authors upon request.

2. † Two-sided p<0.1

* Two-sided p<0.05

** Two-sided p<0.01

*** Two-sided p<0.001

The impact of marijuana use on smoking behavior differs across the two schools. We detect a statistically significant creation function in Sunshine High: a one unit increase in marijuana use increases the odds 62% that adolescent non-smoker will initiate smoking rather than stay as a non-smoker. There was no evidence of a statistically significant endowment function in Sunshine High. On the other hand, the pattern is reversed in Jefferson High with a statistically significant endowment function but a statistically insignificant creation function. Thus, in Jefferson High although marijuana use does not impact respondent's likelihood of smoking initiation, one unit higher marijuana use made smokers 27% more likely to stay as smokers rather than quit smoking at the next time point.

### Simulation results

To understand the magnitude of these effects (e.g., the effect size, or role of these effects in explaining overall substance use rates), we engaged in a small simulation study in which we omitted some of the effects from the SAB model shown in Table 2 and assessed the consequences for the level of substance use behavior in the schools. That is, we changed a particular parameter value from the one estimated in the model to zero, and then simulated the networks and behaviors forward 1000 times. We then assessed the average level of smoking, drinking, and marijuana use in the network at the end of the simulation runs. To save space, we only present the results for Sunshine High; see S2 File for the Jefferson High results, which were similar.

**Fig 3:**
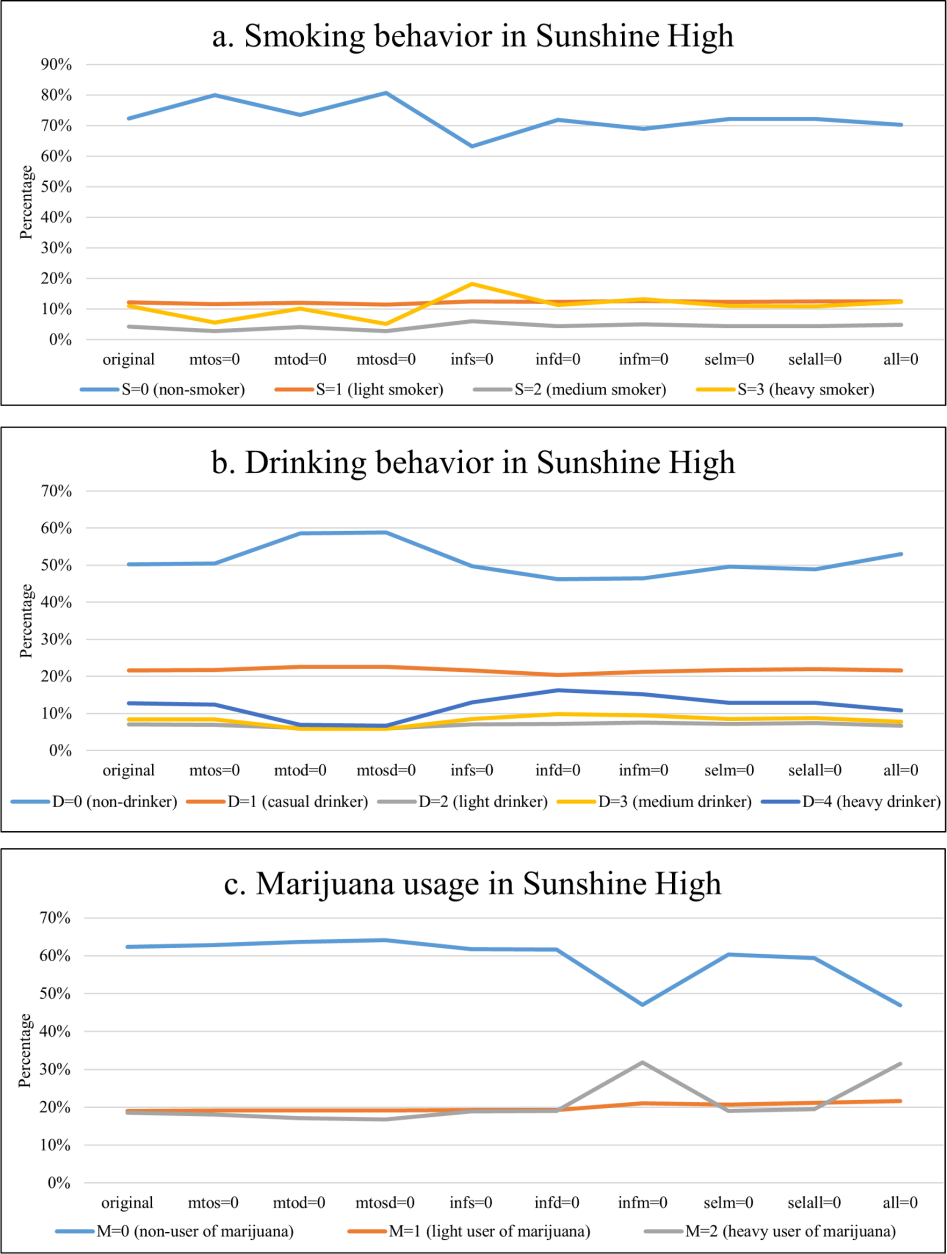
The simulation results of smoking, drinking, and marijuana use levels in Sunshine High under various scenarios setting coefficients to zero. Note: mtos–effect from marijuana use to smoking; mtod–effect from marijuana use to drinking; mtosd–effects from marijuana use to both smoking and drinking; infs–peer influence effect in smoking; infd–peer influence effect in drinking; infm–peer influence effect in marijuana use; selm–peer selection effect in marijuana use; selall–peer selection effects in smoking, drinking, and marijuana use; all–all above effects.

The highest level of smoking is observed when we set to zero the influence effect of friends on smoking behavior, as the percentage of non-smokers drops from 72% in the original model to 63%, and the percentage of heavy-smokers increases from 11% to 18% (Fig 3A). The pattern was similar in Jefferson High, with analogous values of 48% to 42%, and 31% to 35%. This corroborates the findings in previous simulation research that peer influence has a protective effect on smoking [38–39] and drinking [40] adoption. The lowest levels of smoking are observed in the hypothetical scenario in which marijuana use has no effect on one's own smoking behavior, as the percentage of non-smokers rises from 72% to 81%, and the percentage of heavy-smokers decreases from 11% to 5%. The analogous values in Jefferson High were 48% to 54%, and 31% to 25%.

Regarding drinking behavior, we see that the effect of one's own marijuana use is particularly important as setting this effect to zero results in a decrease in drinking behavior (see Fig 3B). In the scenario of no effect of marijuana use on drinking behavior the percentage of non-drinkers rises from 50% to 59% and the percentage of heavy drinkers falls from 13% to 7%. The analogous values in Jefferson High were 35% to 42% and 16% to 10%. It is notable that setting the influence effect of friends' drinking on one's own drinking behavior to zero reduces drinking somewhat (non-drinkers fall to 46% and heavy drinkers rise to 16%). In Jefferson High, the number of heavy drinkers rises from 16% to 20%.

For marijuana usage, very pronounced strong effects are observed for friends' influence (Fig 3C). Setting this influence effect to zero results in a sharp decrease in non-marijuana users from 62% to 47%, and a parallel large increase in heavy users from 19% to 32%. In Jefferson High, the analogous values were 61% to 43% and 18% to 33%.

In sum, when the effect from marijuana use to cigarette (or alcohol) use is turned off, more non-smokers (or non-drinkers) and fewer heavy-smokers (or heavy-drinkers) are expected in both schools. When the peer influence effect with regard to each substance use is turned off, fewer non-users and more heavy-users of each substance are expected in both schools. In the scenarios in which we set other parameters to zero, the simulation results indicated that the substance use distribution was not altered in either school.

## Discussion

Overall, our findings indicate some evidence of sequential substance use, as adolescent marijuana use increased subsequent smoking and drinking behavior in our two school samples. Whereas some existing research has found evidence that marijuana use leads to use of these substances [11, 13, 16, 18, 19], an important contribution of our study was simultaneously taking into account the substance use behavior of adolescents' peer networks and other social processes occurring in networks. We found that marijuana use resulted in more smoking and drinking in both samples. Our findings are partially consistent with Pearson et al. [23], who found that that marijuana users smoked cigarettes more over time. Our findings are suggestive that marijuana use increases both alcohol and cigarette use.

In addition, we made a distinction between whether interdependent substance use going from marijuana to cigarettes and alcohol results in initiation, cessation, or both. We found that marijuana use resulted in drinking initiation in both samples, and smoking initiation in Sunshine High. In contrast, marijuana use decreased the likelihood of smoking cessation in Jefferson High. Previous literature suggests that alcohol use is not a prerequisite for the initiation of marijuana use [14–15] and the effect of alcohol use on the onset of marijuana use has declined while that of marijuana use on the onset of alcohol use has increased since 1965 [14], and our findings are consistent with this prior literature.

Moreover, we tested cross-substance influence effects, which assessed whether the substance use behavior of one's friends on a particular substance affected an individual's own use of the other two substances. We found no evidence that such effects exist in our samples. We did, however, find peer influence effects for each specific substance, which is consistent with multiple past studies [41–44]. Note, however, that whereas one implication is that having more friends who use marijuana, for example, results in greater marijuana use behavior on the part of the individual, another implication is that having more friends who *do not* use marijuana results in *less* marijuana use behavior. This relative symmetry of influence effects is sometimes overlooked when interpreting influence results, and our simulation results confirmed that this influence effect is in fact more likely to have a *negative* effect on substance use behavior. These results are similar to an earlier simulation study that found that increasing the amount of peer influence in two high schools *diminished* school level smoking and drinking behavior [39–40]. These results are consistent with theoretical insights from the *Dynamic Social Impact Theory* [21], which would predict that youth in friendship networks would adopt the same substance use behaviors through peer influence pathways, likely through social proximity and consolidation of youths' attitudes and behaviors in adolescent networks. This highlights that the presumption that influence effects will always increase behavior is not necessarily accurate. In fact, we might expect that the dominant norms in a context will drive the direction of influence effects: in a school with little substance use, the greater number of non-users will push adolescents towards non-use, whereas in a school with high levels of substance adolescents are more likely pushed towards greater use.

Given the complexity of our agent-based network models, we demonstrated the relative magnitude of the effects by combining a small-scale simulation with a strategy in which we constructed hypothetical models that set certain key effects to zero and simulated the networks and behaviors forward. A key finding was that in a simulated world in which one's own marijuana use did not affect smoking or drinking behavior, there would be a notable decrease in overall levels of smoking and alcohol usage in these schools, even controlling for the complexity of these models. We also saw that marijuana use operates as a mechanism between friends' marijuana use and one's own smoking and drinking behavior, as adolescents' use of marijuana is impacted by their friends' marijuana use, and this then affects the adolescent's level of cigarette and alcohol use. Furthermore, one of the strongest effects detected was the influence effect of friends' marijuana usage, as this has a particularly strong relationship to adolescents' own marijuana use. Our findings highlight the importance of understanding interdependence in the use of multiple substances in adolescence, particularly those which operate through peer influence effects within friendship networks.

Another notable finding was that depressive symptoms increased smoking behavior in Jefferson High. This high school has a relatively high average level of substance use compared to Sunshine High. Perhaps in a social milieu with a high average level of drug use, adolescents reporting higher levels of depressive symptoms may be more likely to display higher levels of cigarette smoking as compared to those who report lower level of depressive symptoms, given that past studies link depression and adolescent smoking [45].

There are some limitations to note in this study. First, the time lags between the two sets of waves are not equal (8.8 months vs. 10.9 months apart in Sunshine High and 7.7 months vs. 11.1 months apart in Jefferson High). Although it is preferable to have equal time periods, we performed a post hoc time heterogeneity test [30] to ensure that the co-evolution of substance use behaviors and friendship networks was not significantly different across the three waves, or two time periods. Second, our SAB model specification is data intensive and can only be estimated for the two large schools among the 16 saturated schools in Add Health which are feasible for this type of analysis. This limits generalizability and does not allow assessing why the interdependent effect from marijuana use to smoking is different across the two schools. Third, we had indirect information about marijuana use at time one, for a large percentage of the sample. Using this indirect information allowed us to avoid discarding a large amount of information at *t*_*1*,_ however with a relatively small (less than 10%) amount of potentially misclassified cases. Fourth, while the data are relatively old, we are aware of no evidence that the mechanisms of in person friendship formation, as captured in these Add Health network data, have changed significantly since the mid-nineties. In the current study, friendship networks were constructed through name generator items instead of real-time communication technology such as cell phone use. While future studies are needed to leverage existing technology such as cell phone usage for collecting adolescent social network data, these in person network data are likely still meaningful. Moreover, research suggests that cell phones help reinforce and reproduce existing social roles and structures rather than alter them [46–47]. That said, future studies are needed to collect nationally representative contemporary data from US adolescents and investigate how the findings herein would be different if such technology was considered.

### Implications

Our findings have important implications for future studies. First, our findings suggest both feasibility and merit in exploring concurrent or sequential substance use behaviors across multiple time periods. Interdependence in substance use should be studied within one single model framework with multiple simultaneous on-going processes (e.g., peer selection, peer influence, parental influence, and etc.) to reduce the risk of over-estimation of each process due to the autocorrelation among them. Second, further explication of the interdependent effects from marijuana use to smoking and drinking is a useful direction for future research. Third, given smoking rates among adolescent youth have decreased significantly since the mid-1990s, more recent data are required to test whether our findings from these two Add Health large schools can be replicated in future research.

Our findings also have practical implications for health behavior change interventions targeting adolescent substance use. Studies have found evidence of a protective effect of social network ties for adolescent substance use [48]. Moreover, other research indicates that social networks can be leveraged for health behavior change interventions and may even be superior to non-network based interventions [49]. Peer network based interventions targeting adolescent substance use might address the possibility that marijuana use increases alcohol and cigarette use. One approach to do this would be to disseminate tailored messages through adolescent peer networks to modify norms favoring the concurrent use of these substances, and therefore alter peer influences condoning the use of one of these substances or the concurrent use of two these substances. Such messages could act as cues to action to halt peer influences facilitating the progression from use of one substance to using both, concurrently.

Lastly, a policy-relevant implication of our finding that marijuana use appears to lead to more cigarette and alcohol use is that there may be unintended consequences for adolescent substance use from the legalization of marijuana in states. If such legalization leads to greater marijuana use among adolescents, our results suggest that more cigarette smoking and alcohol drinking behavior among adolescents might occur concurrently. This is a possibility that has received some research attention [50] and should be given more consideration in future work.

### Conclusions

Our findings suggest that marijuana use leads to the usage of other substances among the adolescent youth under study. More specifically, it is related to the initiation (i.e., uptake process) of drinking behavior. It might be associated with initiation of (i.e., uptake process) of smoking behavior, when the average substance use level in the school context is low (e.g., in Sunshine High). It also may stop smokers from quitting when the average substance use level is high (e.g., in Jefferson High).

## Supporting information

S1 Table Stochastic actor-based models of friendship networks and substance use of cigarettes, alcohol, and marijuana with friends' average smoking/drinking/marijuana use level effects. (PDF)

S1 File Goodness-of-fit (GOF) for SAB models. (PDF)

S2 File The simulation results of smoking, drinking, and marijuana use levels in Jefferson High under various conditions. (PDF)
