# Supplementary material for: The interdependence of cigarette, alcohol, and marijuana use in the context of school-based social networks
Source: PLoS One. 2018 Jul 20;13(7):e0200904. doi: 10.1371/journal.pone.0200904 (PMC6054419; doi:10.1371/journal.pone.0200904)
Supplement: S1 Table — (PDF) [file pone.0200904.s001.pdf]

S1 Table. Stochastic Actor-Based models of friendship networks and substance use of cigarettes, alcohol, and marijuana with friends' average smoking/drinking/marijuana use level effects

Effect name	Sunshine High	Jefferson High
Smoking behavior	beta (s.e.)	beta (s.e.)
Rate smoking behavior (period 1)	13.18** (4.86)	9.14*** (1.57)
Rate smoking behavior (period 2)	21.31*** (3.28)	13.84*** (3.42)
Smoking behavior linear shape	-2.51*** (0.11)	-2.16*** (0.10)
Smoking behavior quadratic shape	0.73*** (0.04)	0.70*** (0.06)
In-degree	0.01 (0.01)	0.01 (0.01)
Smoking behavior peer influence	0.54*** (0.13)	0.77*** (0.15)
Parental support	-0.13 (0.19)	-0.02 (0.14)
Parental monitoring	-0.05 (0.26)	-0.05 (0.22)
Black	-0.33** (0.12)	-
Latino	-0.13* (0.05)	-
Depressive symptoms	0.07 (0.06)	0.12* (0.05)
Drinking behavior	0.05† (0.03)	0.02 (0.06)
Number of friends who drank	0.01 (0.02)	0.00 (0.01)
Marijuana use	0.22*** (0.06)	0.14* (0.06)
Number of friends who used marijuana	0.02 (0.05)	-0.02 (0.03)
Drinking behavior		
Rate drinking behavior (period 1)	15.86*** (1.68)	9.94*** (0.94)
Rate drinking behavior (period 2)	16.83*** (2.24)	13.21* (5.68)
Drinking behavior linear shape	-1.38*** (0.07)	-1.01*** (0.11)
Drinking behavior quadratic shape	0.27*** (0.03)	0.20*** (0.02)
In-degree	0.01	0.01

	(0.01)	(0.01)
Drinking behavior peer influence	0.28** (0.12)	0.38* (0.16)
Parental support	-0.06 (0.06)	-0.07 (0.06)
Parental monitoring	-0.19 (0.20)	-0.47** (0.17)
Black	-0.11* (0.04)	-
Latino	0.07† (0.04)	-
Depressive symptoms	0.06* (0.03)	0.02 (0.03)
Smoking behavior	0.03 (0.06)	0.01 (0.02)
Number of friends who smoked	0.01 (0.01)	0.01 (0.01)
Marijuana use	0.20*** (0.05)	0.15** (0.05)
Number of friends who used marijuana	0.01 (0.02)	-0.02 (0.01)
Marijuana use		
Rate marijuana use (period 1)	2.68*** (0.50)	2.42*** (0.34)
Rate marijuana use (period 2)	4.97*** (0.57)	4.83*** (0.83)
Marijuana use linear shape	-2.48*** (0.29)	-2.14*** (0.32)
Marijuana use quadratic shape	1.13*** (0.13)	1.06*** (0.09)
In-degree	0.03 (0.05)	0.02 (0.02)
Marijuana use peer influence	1.43** (0.38)	1.32*** (0.49)
Parental support	-0.03 (0.23)	0.12 (0.19)
Parental monitoring	-0.34 (0.46)	0.00 (0.46)
Black	0.17 (0.11)	-
Latino	0.07 (0.09)	-
Depressive symptoms	-0.08 (0.08)	0.12 (0.12)
Smoking behavior	0.07 (0.11)	0.05 (0.06)

Number of friends who smoked	0.02<br>(0.06)	0.03<br>(0.05)
Drinking behavior	0.12<br>(0.08)	0.06<br>(0.08)
Number of friends who drank	-0.04<br>(0.04)	-0.04<br>(0.04)
Friendship network dynamics	beta<br>(s.e.)	beta<br>(s.e.)
Friendship rate (period 1)	16.04***<br>(0.81)	18.02***<br>(1.40)
Friendship rate (period 2)	7.75***<br>(0.78)	11.75***<br>(0.93)
Out-degree (density)	-4.15***<br>(0.18)	-2.14***<br>(0.40)
Reciprocity	3.11***<br>(0.16)	2.57***<br>(0.11)
Transitive triplets	0.71**<br>(0.22)	0.62***<br>(0.05)
3-cycles	-0.29<br>(0.40)	-0.42***<br>(0.12)
Out-degree – popularity	-0.32***<br>(0.08)	-0.19***<br>(0.04)
In-degree – popularity	-0.05**<br>(0.02)	-0.06**<br>(0.02)
Out-out degree^(1/2) assortativity	-0.07*<br>(0.03)	-0.12***<br>(0.02)
In-in degree^(1/2) assortativity	0.48***<br>(0.08)	0.37***<br>(0.07)
Race similarity	1.18***<br>(0.08)	-
Gender similarity	0.29***<br>(0.04)	0.23***<br>(0.06)
Grade similarity	0.62***<br>(0.05)	0.57***<br>(0.05)
Parental education similarity	0.09**<br>(0.03)	0.05<br>(0.05)
Smoking similarity (peer selection)	0.01<br>(0.08)	0.24*<br>(0.10)
Drinking similarity (peer selection)	0.12*<br>(0.05)	0.13**<br>(0.05)
Marijuana use similarity (peer selection)	0.27***<br>(0.07)	0.22*<br>(0.09)
Limited nomination ego	-0.84***<br>(0.25)	-1.38***<br>(0.21)

† Two-sided p<0.1; * Two-sided p<0.05; ** Two-sided p<0.01; *** Two-sided p<0.001
