# Supplementary material for: The interdependence of cigarette, alcohol, and marijuana use in the context of school-based social networks
Source: PLoS One. 2018 Jul 20;13(7):e0200904. doi: 10.1371/journal.pone.0200904 (PMC6054419; doi:10.1371/journal.pone.0200904)
Supplement: S1 File — (PDF) [file pone.0200904.s002.pdf]

S1 File. Goodness-of-fit (GOF) for SAB models

Goodness-of-Fit (GOF) testing has gradually become the new standard in the field of SAB modeling (Snijders, 2014). Lospinoso (2012) proposed to use the Monte Carlo Mahalanobis Distance test for SAB models, which is implemented in the RSiena package (Ripley et al., 2017). A *p*-value is reported to test whether the average values of the auxiliary network statistics (e.g., distribution of in-degree, out-degree, geodesic distance, and triad census) over *m* (1000 by default) simulation runs are close to the values observed in the data. Plotting functions can be used to diagnose bad fit (Ripley et al., 2017; Lospinoso & Snijders, 2011).

We assessed the appropriateness of our model specification by assessing key network statistics in the schools during the final time point (wave 3). Figure S2 shows the GOF testing results of estimated SAB models for Sunshine High. The *p*-values of the four auxiliary statistics are greater than 0.05. In other words, the null hypothesis that our SAB models could reproduce several key network and behavior statistics at the final time point is not rejected. The GOF testing results of the other SAB models are available from the authors upon request.

References

Barabási AL, Albert R. Emergence of scaling in random networks. *Science* 1999; 286: 509-12.
Lospinoso JA. *Statistical Models for Social Network Dynamics*. PhD thesis, University of Oxford, UK, 2012.
Lospinoso JA, Snijders TAB. *Goodness of Fit for Social Network Dynamics*. Presentation at the Sunbelt XXXI, St. Pete's Beach, FL, 2011.
Newman MEJ. Assortative mixing in networks. *Physical Review Letters* 2002; 89: 208701.
Ripley RM, Snijders TAB, Boda Z, Vörös A, Preciado P. *Manual for SIENA version 4.0 (version May 12, 2017)*. Oxford: University of Oxford, Department of Statistics, Nuffield College; 2017. Available at: http://www.stats.ox.ac.uk/siena/. Accessed July 18, 2017.
Snijders TAB. *Siena Advanced Users' Meeting 2014*. Presentation at the Sunbelt XXXIV, St. Petersberg, Florida, 2014.

Goodness of Fit of Outdegree Distribution Wave 3

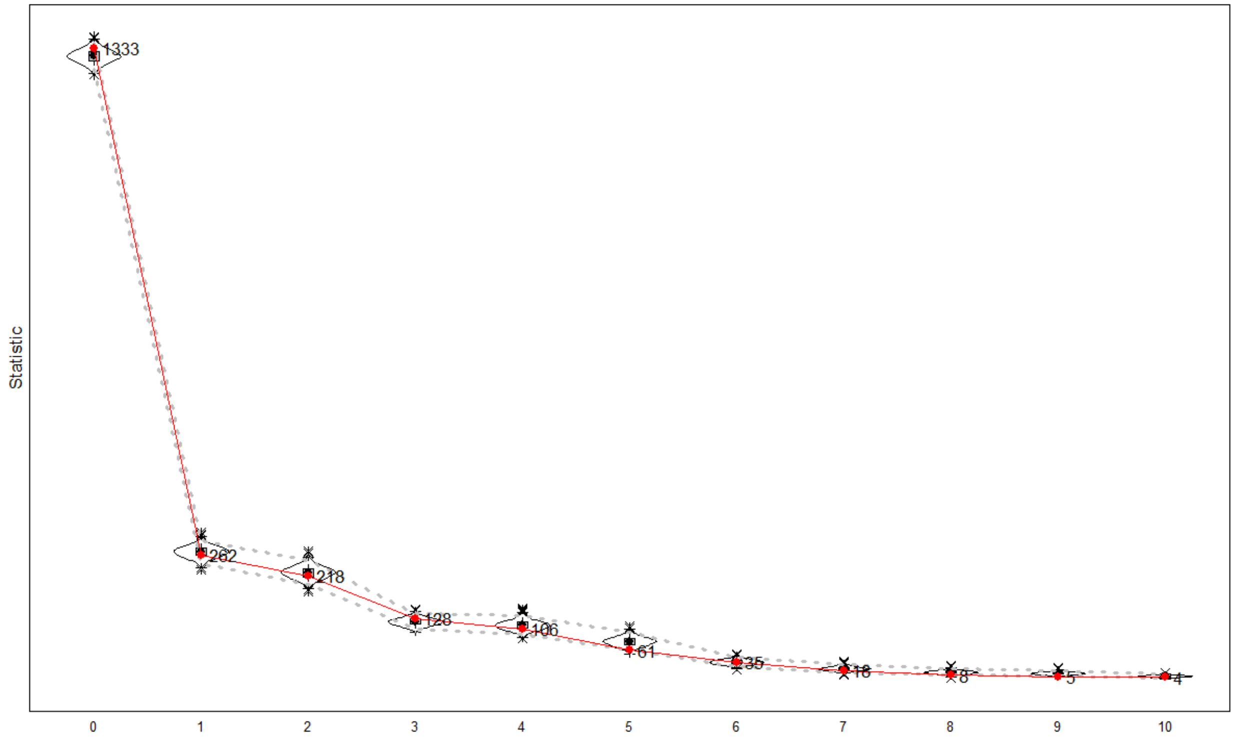

p: 0.354

Goodness of Fit of Indegree Distribution Wave 3

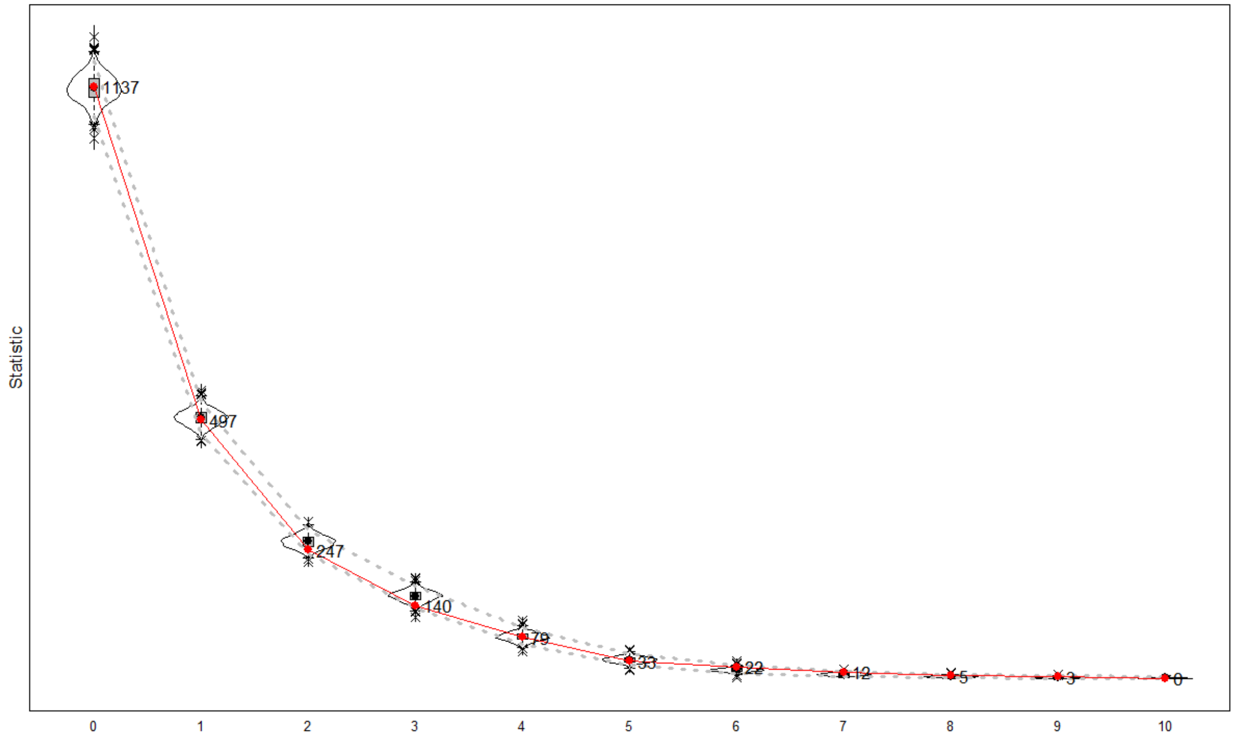

p: 0.194

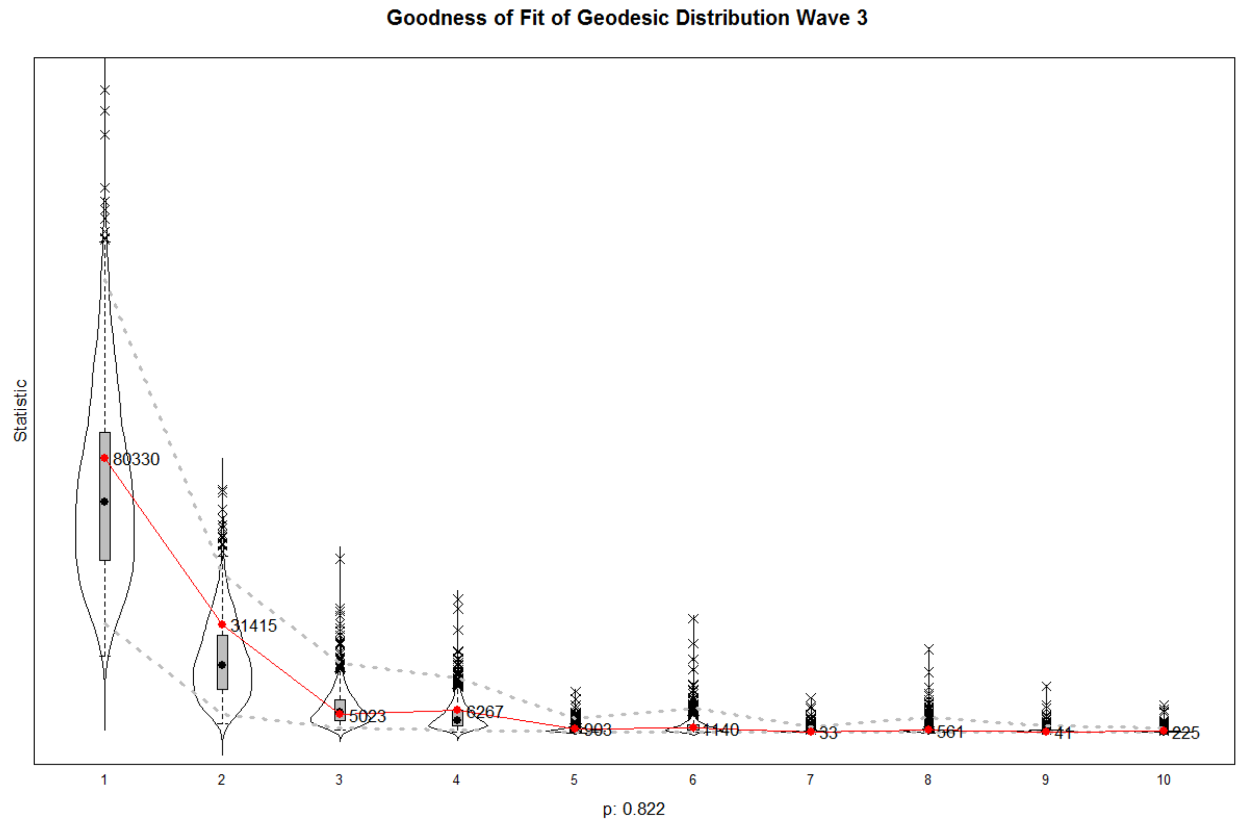

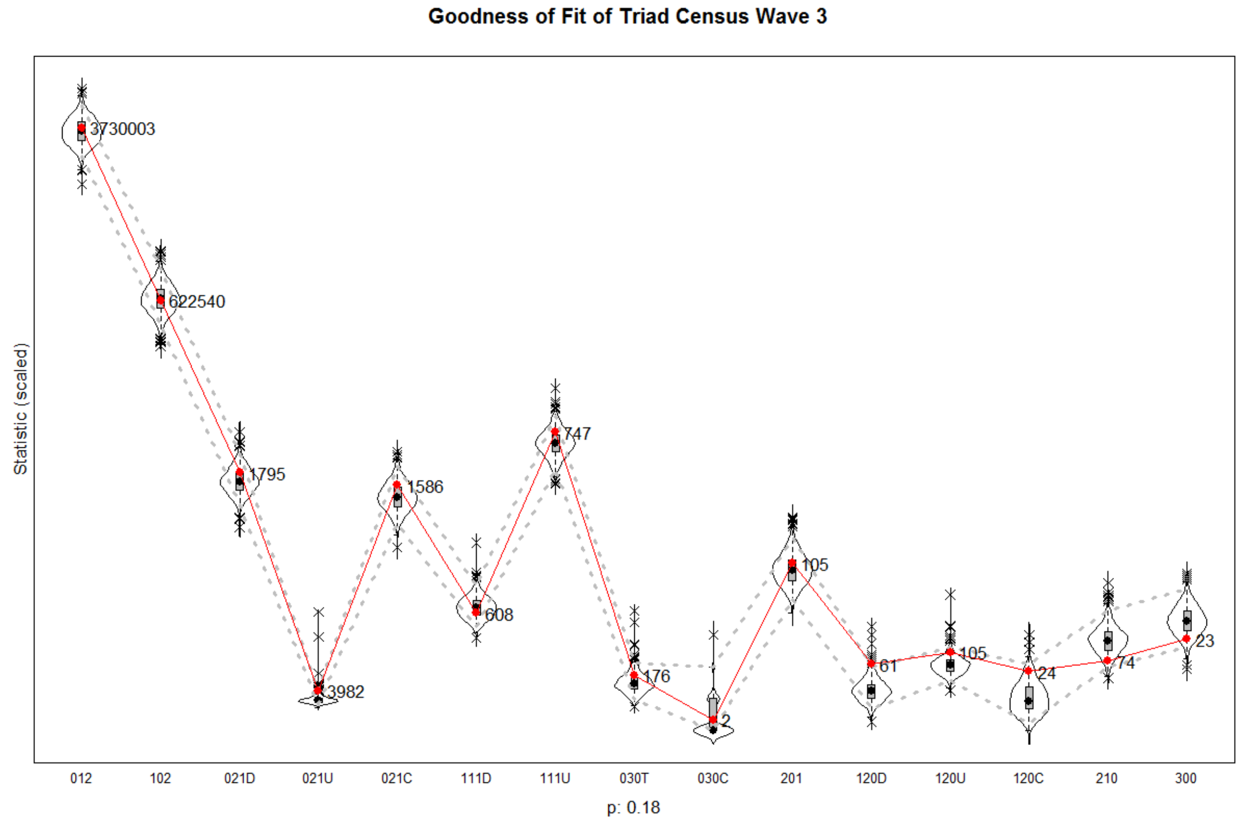

Figure. Goodness-of-fit testing of SAB model for Sunshine High
