# Supplementary material for: The interdependence of cigarette, alcohol, and marijuana use in the context of school-based social networks
Source: PLoS One. 2018 Jul 20;13(7):e0200904. doi: 10.1371/journal.pone.0200904 (PMC6054419; doi:10.1371/journal.pone.0200904)
Supplement: S2 File — (PDF) [file pone.0200904.s003.pdf]

S2 File. The simulation results of smoking, drinking, and marijuana use levels in Jefferson High under various conditions

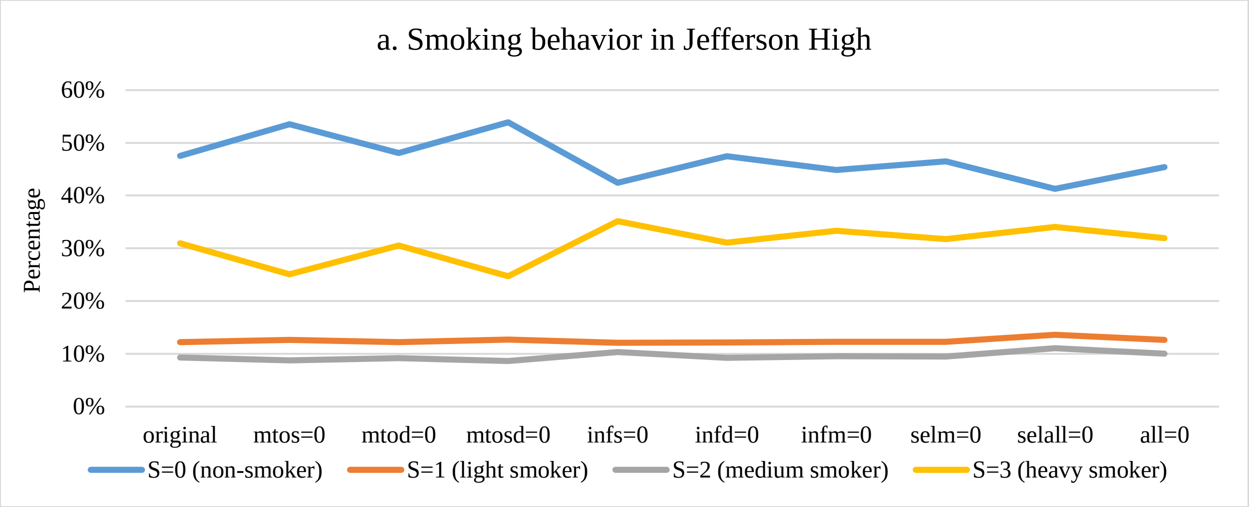

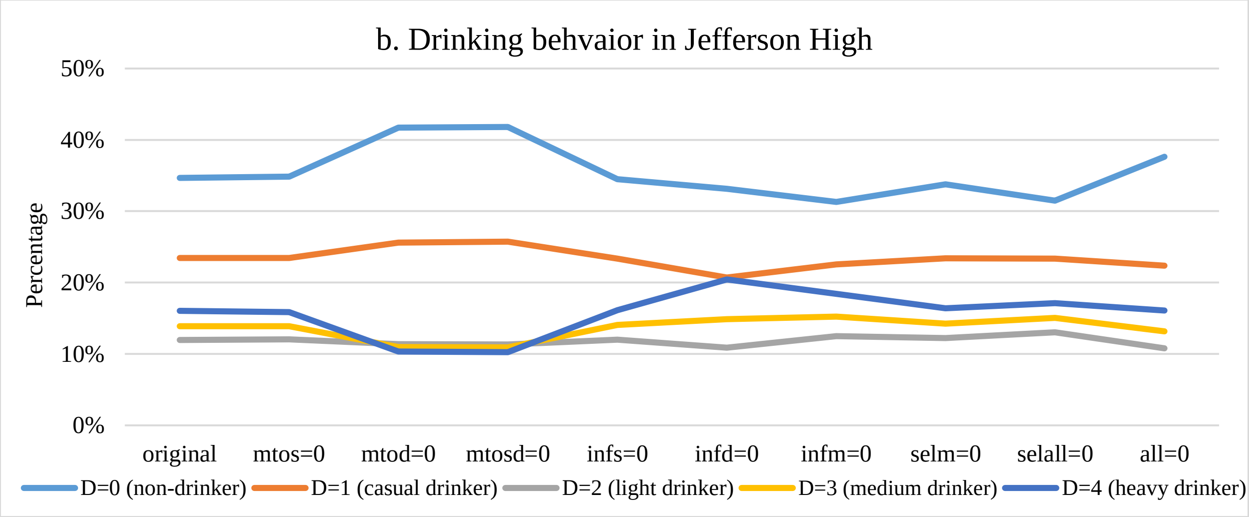

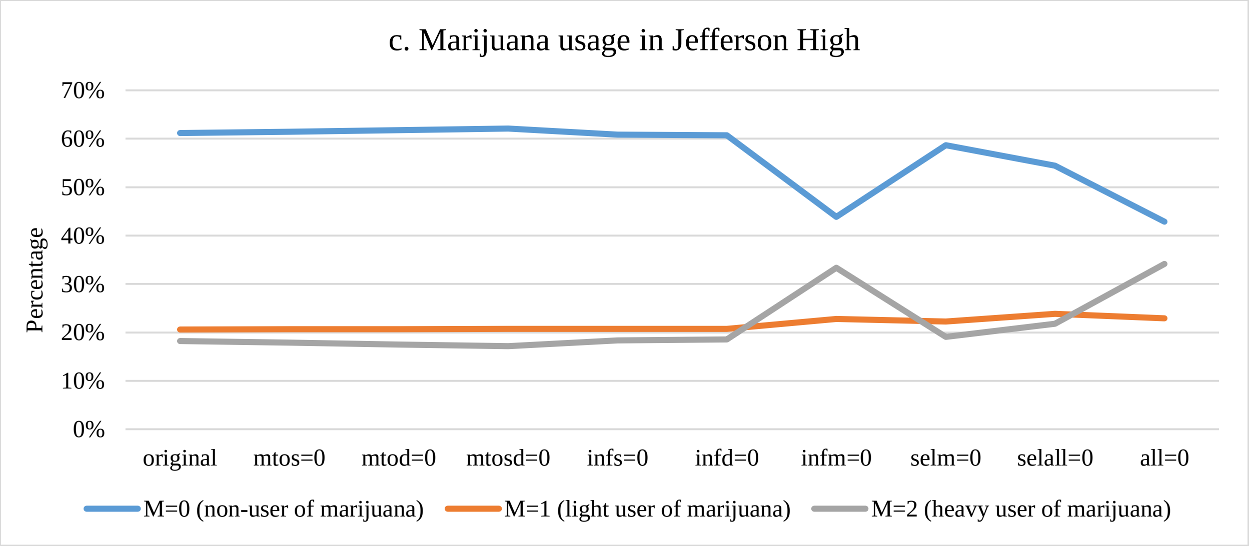

Note: mtos – effect from marijuana use to smoking; mtod – effect from marijuana use to drinking; mtosd – effects from marijuana use to both smoking and drinking; infs – peer influence effect in smoking; infd – peer influence effect in drinking; infm – peer influence effect in marijuana use; selm – peer selection effect in marijuana use; selall – peer selection effects in smoking, drinking, and marijuana use; all – all above effects
